# A comparative transcriptomic analysis reveals the core genetic components of salt and osmotic stress responses in *Braya humilis*

**DOI:** 10.1371/journal.pone.0183778

**Published:** 2017-08-31

**Authors:** Pengshan Zhao, Lirong Wang, Xin Zhao, Guoxiong Chen, Xiao-Fei Ma

**Affiliations:** 1 Key Laboratory of Stress Physiology and Ecology in Cold and Arid Regions, Gansu Province, Northwest Institute of Eco-Environment and Resources, Chinese Academy of Sciences, Lanzhou, P. R. China; 2 Shapotou Desert Research and Experimental Station, Northwest Institute of Eco-Environment and Resources, Chinese Academy of Sciences, Lanzhou, P. R. China; 3 College of Forestry, Gansu Agricultural University, Lanzhou, Gansu Province, P. R. China; Institute of Botany Chinese Academy of Sciences, CHINA

## Abstract

*Braya humilis* is a member of the Euclidieae tribe within the family Brassicaceae. This species exhibits a broad range of adaptations to different climatic zones and latitudes as it has a distribution that ranges from northern Asia to the arctic-alpine regions of northern North America. In China, *B*. *humilis* is mainly found on the Qinghai—Tibetan Plateau (QTP) and in adjacent arid regions. In this study, we sequenced a sample from an arid region adjacent to the QTP using the Illumina platform generating a total of 46,485 highly accurate unigenes, of which 78.41% were annotated by BLASTing versus public protein databases. The *B*. *humilis* transcriptome is characterized by a high level of sequence conservation compared with its close relative, *Arabidopsis thaliana*. We also used reciprocal blast to identify shared orthologous genes between *B*. *humilis* and four other sequenced Brassicaceae species (i.e. *A*. *thaliana*, *A*. *lyrata*, *Capsella rubella*, and *Thellungiella parvula*). To enable precise characterization of orthologous genes, the early-diverging basal angiosperm *Amborella trichopoda* was also included. A total of 6,689 orthologous genes were identified before stricter criteria for the determination of e-values, amino acid hit lengths, and identity values was applied to further reduce this list. This led to a final list of 381 core orthologous genes for *B*. *humilis*; 39 out of these genes are involved in salt and osmotic stress responses and estimations of nonsynonymous/synonymous substitution ratios for this species and *A*. *thaliana* orthologs show that these genes are under purifying selection in *B*. *humilis*. Expression of six genes was detected in *B*. *humilis* seedlings under salt and osmotic stress treatments. Comparable expression patterns to their counterparts in *Arabidopsis* suggest that these orthologous genes are both sequence and functional conservation. The results of this study demonstrate that the environmental adaptations of *B*. *humilis* are mainly the results of preexisting genetic components. Future work will be required to characterize the expression patterns of these orthologous genes in natural populations and will provide further insights into the adaptive mechanisms underlying the wide range of *B*. *humilis* adaptations.

## Introduction

The Brassicaceae family is well-known because it includes the model plant *Arabidopsis thaliana* as well as numerous other economically important crop species, including *Brassica spp*. and pennycress (*Thlaspi arvens*e), and the extremophyte *Thellungiella parvula* (also known as *Schrenkiella parvula*) [1–4]. Previous comparative studies have revealed a high level of global sequence conservation within the Brassicaceae family [2–5]; 43.3% of pennycress transcripts, for example, have homologous genes with higher than 70% positive match percentages when compared to the *A*. *thaliana*, *A*. *lyrata*, *Capsella rubella*, *Brassica rapa*, and *T*. *halophila* protein databases, while some genes putatively involved in flowering and glucosinolate metabolism have also been identified in the pennycress transcriptome [2]. Extensive sequence conservation and synteny have also been identified between *T*. *parvula* and the abiotic stress-sensitive species *A*. *thaliana* [3,4]. These studies highlight the fact that genetic elements underlying speciation and adaptation are conserved and suggest that comparative study is one effective approach to uncover the putative genes controlling important traits and environment adaptations seen in the Brassicaceae family.

*Braya humilis* is a member of the family Brassicaceae within the tribe Euclidieae. This species is distributed across the arctic-alpine regions of northern North America and into northern Asia, including Russia and China, south to the Himalayas, and west to Afghanistan, Pakistan, and Central Asia [6]. Of these regions, Central Asia has been proposed as the primary center of diversity for the *Braya* genus [7] The species *B*. *humilis* comprises polyploids that have a base chromosome number x = 14/16 and a total chromosome number that ranges between 14 and 70 [7]; the results of our survey work shows that this species is mainly distributed across the Qinghai-Tibetan Plateau (QTP) and in the adjacent arid regions of China, and the phenotypes of *B*. *humilis*, including flower size, color, and leaf morphology, are variable in these different areas, consistent with the description presented by Zhou et al. [8]. The diversity of growth forms and ecological adaptations seen in this species provides a clear incentive to unravel the mechanisms of these adaptations at the molecular level.

We performed *de novo* transcriptome sequencing of a sample of *B*. *humilis* from the arid region adjacent to the QTP in this study using the Illumina HiSeq2000 platform. Reciprocal blast was performed to identify shared orthologous genes between *B*. *humilis* and four other sequenced Brassicaceae species (i.e. *A*. *thaliana*, *A*. *lyrata*, *Capsella rubella*, and *T*. *parvula*) as well as the early-diverging basal angiosperm *Amborella trichopoda*. Gene ontology (GO) enrichment, nonsynonymous to synonymous substitution (Ka/Ks) ratio analyses, and transcriptome profiles of *A*. *thaliana* orthologs were also used to provide more insights into the function and evolutionary pattern of core salt and osmotic stress response genes in the study species.

## Materials and methods

### Ethics statement

The species *B*. *humilis* is widely distributed across northern China and is not listed as either endangered or protected. Thus, no specific permits were required to collect seeds for this study.

### Plant materials, RNA extraction, library construction, and RNA-seq

Fresh seeds of *B*. *humilis* were sampled in Yuzhong County (35°56'21"N, 104°08'13"E, Altitude: 1805–1979 m), Gansu Province, northwestern China, Where the average annual precipitation is 318 mm. All seeds were stored in our laboratory for one month after harvesting. Since the local habitat is characterized by an arid climate with low annual precipitation during the late spring and summer, we mainly focused on the early growth stage of *B*. *humilis* in this study; thus, the aboveground parts of seedlings were sampled 21 days after stratification and total RNA was extracted using a Plant total RNA Kit (#DP432, TIANGEN, Beijing, China), while the concentration and quality of the sample was determined using 1% agarose gel electrophoresis in a NanoDrop 2000^™^ micro-volume spectrophotometer (Thermo Scientific, Waltham, MA, USA) and with an Agilent 2100 Bioanalyzer (Agilent Technologies, Santa Clara, CA, USA). We prepared a complementary DNA library following the protocol outlined by Zhao et al [9]; in short, purified poly-A mRNA was fragmented and converted to cDNA via reverse transcription and random priming before fragments were ligated to sequencing adaptors after being blunt-ended, size-selected for an insert of 200 base pairs (bp), and then enriched using PCR with specific adaptor primers. A library was then sequenced using the Illumina HiSeq^™^ 2000 platform and 101 bp paired-end reads. This approach generated a total of 21.64 million reads, 80% of which were above Q30. All the sequence data generated in this study were deposited in the National Center for Biotechnology Information (NCBI) Short Read Archive under accession number SRS1143215.

### Assembly and functional annotation

We assembled the transcriptome of *B*. *humilis* following the protocol outlined by Zhao et al. [9]. Unigenes were used as a reference for subsequent functional annotation and comparative analyses. We performed the first of these analyses by aligning unigenes with public protein databases using the Blast program, setting an e-value cutoff less than 1e-5, while gene ontology (GO) analysis was carried out using the Blast2GO platform [10]. Reads per kilobase per million reads (RPKM) for each unigene were calculated to determine its expression profile [11].

### Comparative transcriptomic analysis

We downloaded the proteome of *A*. *thaliana* (35,386 peptides) from the TAIR10 release (www.arabidopsis.org) and compared it with assembled *B*. *humilis* unigenes using the Blastx program, setting an e-value cutoff of 1e-10. The best hit from each unigene was then subject to further statistical analyses using the software R, following the protocol of Dorn et al. [2].

Protein sequences for *A*. *lyrata* [12], *C*. *rubella* [13], *T*. *parvula* [3,4], and *A*. *trichopoda* [14,15] were obtained from Phytozome v9.1 (www.phytozome.net). A tBlastn comparison was performed between *A*. *thaliana* peptides and *B*. *humilis* unigenes, and the reciprocal best hits (RBHs) between the results from this comparison and those from Blastx were used as pairs of putative orthologs between these two species. We then performed three pairwise Blastp comparisons (e-value set to less than 1e-10) to screen RBHs between *A*. *thaliana* and another set of three Brassicaceae species, while RBHs between *A*. *thaliana* and *A*. *trichopoda* were obtained using an e-value threshold of 1e-5. Orthologous genes were screened following the protocol described by Golicz et al. [16]; thus, genes from the six species (i.e. *B*. *humilis*, *A*. *thaliana*, *A*. *lyrata*, *C*. *rubella*, *T*. *parvula*, and *A*. *trichopoda*) were isolated from the five RBH results using the ‘VennDiagram’ function in R [17], and those conserved in all six species were subject to further analysis and annotation. GO annotation was then performed for the *A*. *thaliana* genes that corresponded with those shared in the other five species using the default settings in Classification SuperViewer [18]. The enrichment cutoff for *p*-values was defined as less than 0.01 for both normalized class scores and absolute values, while the Kyoto Encyclopedia of Genes and Genomes (KEGG) pathway enrichment was analyzed using the Plant GeneSet Enrichment Analysis Toolkit (PlantGSEA) program [19]. Statistical significance was examined using the Fisher test and multi-test adjustment method was Yekutieli (FDR under dependency). The cutoff FDR value was 0.05.

### Ka/Ks analysis

The coding and untranslated regions of *B*. *humilis* unigenes were predicted based on non-redundant protein (Nr) and Swiss-Prot annotations. Nucleic acid and protein sequences of orthologous genes from *B*. *humilis* and *A*. *thaliana* were used to generate estimates for Ka/Ks ratios using the PAL2NAL platform (http://www.bork.embl.de/pal2nal/index.cgi?example=Yes#RunP2N, [20]).

### Verification of assembled unigenes and quantitative RT-PCR

We isolated leaf total RNA and then used 0.5 μg to synthesize cDNA using a Transcriptor First Strand cDNA Synthesis Kit (Roche, Germany). Primers for verification and qRT-PCR were designed online using the IDT platform (https://www.idtdna.com/pages/scitools). Seven-day-old seedlings were subject to three treatments, water, 150 mM NaCl or 300 mM mannitol, for three hours before being sampled for total RNA extraction, following the protocol outlined by Zhao et al. [9] for RT-PCR and qRT-PCR in order to verify the quality of assembled unigenes and to measure gene expression. The expression levels of core salt and osmotic stress-responsive genes were normalized relative to *T2_36132* (*Elongation factor*, [21]), two biological replicates were performed, and each RNA sample was tested in three times.

## Results and discussion

### RNA-seq and *de novo* assembly

**Fig 1:**
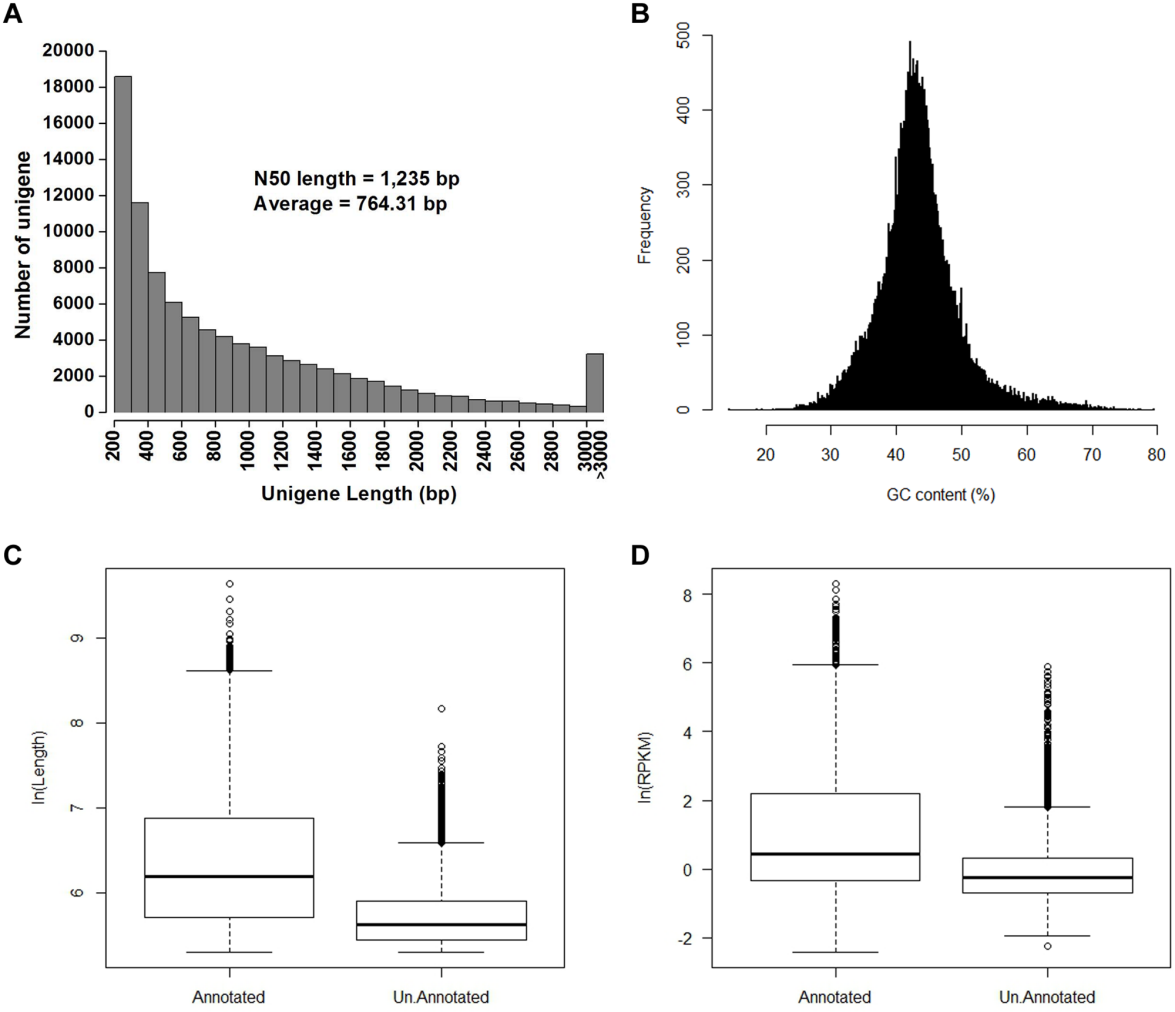
Analyses of the *B*. *humilis* trancriptome. (A) Histogram to show the length distribution of assembled unigenes. (B) Distribution of GC content in unigenes. (C) Length distribution of annotated and un-annotated unigenes inferred using ln(Length). (D) Expression levels of annotated and un-annotated unigenes inferred using ln(RPKM).

We isolated total RNA from the aerial sections of 21-day-old seedlings, constructing a library that was then sequenced using Illumina paired-end technology, leading to the generation of approximately 21.6 Mb of clean reads after stringent quality checks and data filtering. Trimmed reads were then assembled using the Trinity program [22], generating a total of 46,485 unigenes with a mean length of 764 bp (Fig 1A and S1 Table). Results shows that the average GC content of these unigenes is 43.4%, and between 35% and 45% in approximately 68% of unigenes (Fig 1B). In order to further evaluate assembly quality, 24 unigenes were randomly selected and subject to RT-PCR detection; data show that 23 out of 24 were successfully amplified (S1 Fig), suggesting that our transcriptome assembly is highly accurate.

### Annotation and functional characterization of *B*. *humilis* unigenes

All of the unigenes generated in this study were aligned to the NCBI non-redundant protein (Nr), the Swiss-Prot protein, and the Clusters of Orthologous Groups of proteins (COG) databases at thresholds less than 1e-5 (Table 1). The results of these comparisons show that 36,236 (77.95%) and 26,083 (56.11%) of the 46,485 total unigenes were significantly matched with the deposits in the Nr and Swiss-Prot protein databases, respectively. Approximately 62.72% of unigenes (22,726) were mapped to known plant genes with best hits (e-value<1e-50; S2A Fig), while 58.21% (21,093) can hit deposited sequences at similarity levels greater than 80% (S2B Fig).

**Table 1:** Summary of unigene annotations within the *B*. *humilis* transcriptome.

Annotation databases	Unigene	Percentage (%)
Nr	**36,236**	**77.95**
Swiss-Prot	**26,083**	**56.11**
GO	**32,155**	**69.17**
COG	**11,010**	**23.69**
KEGG	**8,431**	**18.14**
Total	**36,451**	**78.41**

GO terms associated with cellular component, molecular function, or biological process (BP) terms were then assigned to each *B*. *humilis* unigene (S2 Table). Results show significant hits in public databases for 69.17% of unigenes (32,155), while 28,434 were assigned more than one GO term. The five most commonly assigned BP GO terms were ‘oxidation-reduction process’ (1,849 unigenes), ‘regulation of transcription’ (1,731 unigenes), ‘response to salt stress’ (1,456 unigenes), ‘response to cadmium ion’ (1,353 unigenes), and ‘protein phosphorylation’ (1,296 unigenes).

It is nevertheless noteworthy that approximately 22% of the unigenes identified in this study were not annotated by our annotation pipeline. Results show that the average lengths of these unigenes were markedly shorter than those of annotated sequences (Fig 1C; p-value < 2.2e-16), while their expression levels, inferred using RPKM, were also much lower (Fig 1D; p-value < 2.2e-16). These results are consistent with our previous finding that both read length and sequencing depth limit transcriptome annotation percentage [9].

### Comparative transcriptomics of *B*. *humilis* and other Brassicaceae species

**Fig 2:**
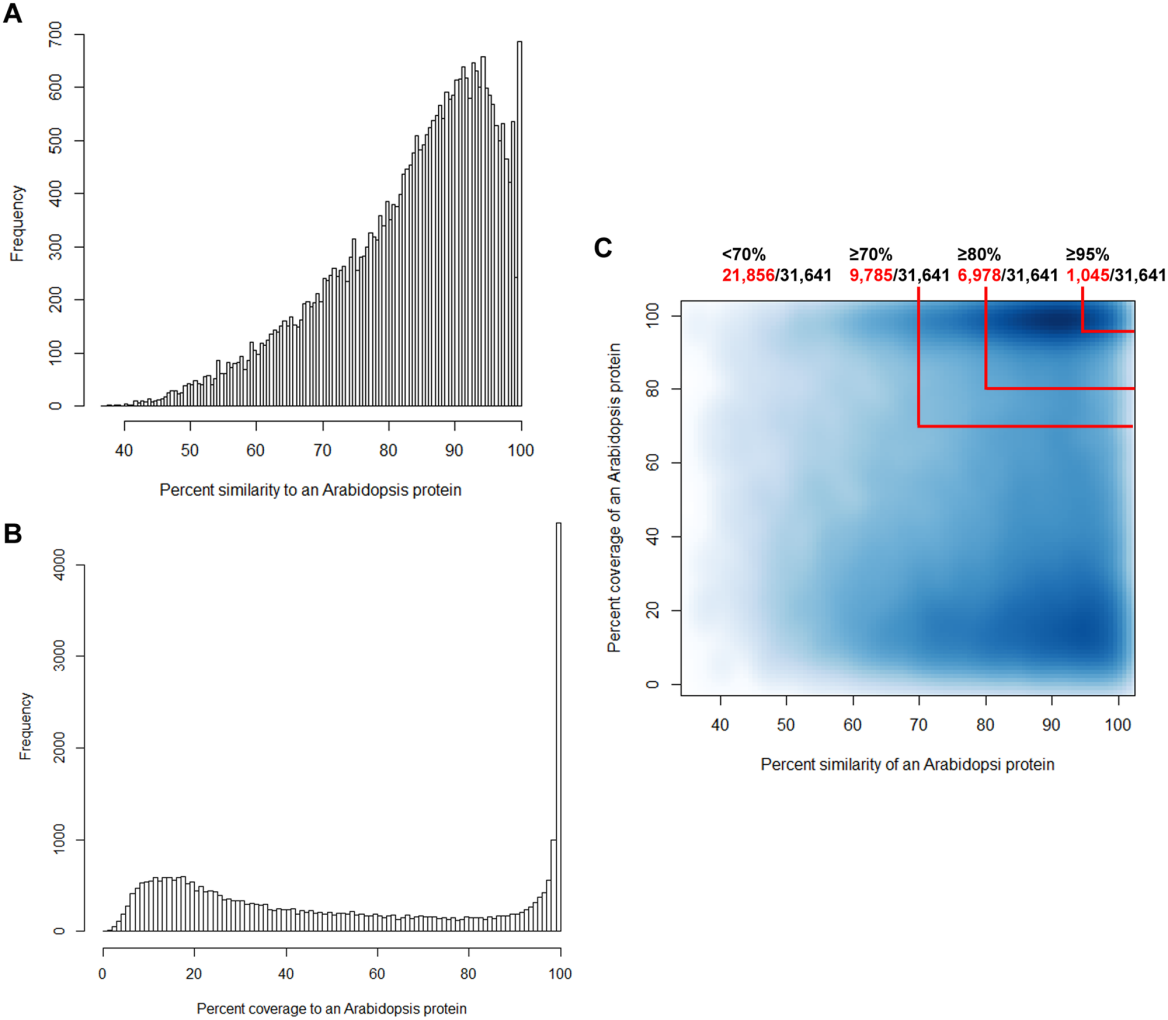
Results of comparative trancriptome analyses between *B*. *humilis* unigenes and *A*. *thaliana* proteins. (A) Frequency of protein identity values for *B*. *humilis* unigenes versus *A*. *thaliana* proteins. (B) Frequency of the percentage coverage of each *B*. *humilis* unigene versus corresponding *A*. *thaliana* protein. Percentage coverage was calculated by dividing protein length by longest positive hit. (C) Smoothed color density map. The percentage similarity (A) and coverage (B) for each *B*. *humilis* unigene versus an *A*. *thaliana* protein were plotted using the ‘smoothScatter’ package in R [17]. *B*. *humilis* unigenes with 70%, 80%, and 95% sequence identity and coverage are shown with red lines, and all statistical analyses were performed following the protocol in Dorn et al. [2].

Molecular data suggest the presence of three major lineages within the family Brassicaceae [23,24]. In terms of species content, *B*. *humilis* is a member of lineage three of the tribe Euclidieae, while *A*. *thaliana*, *A*. *lyrata*, and *C*. *rubella* are all included in lineage one, and *T*. *parvula* is a member of lineage two [23–26]. We therefore performed a further Blastx comparison to measure the percentages of positive sequence similarity and coverage to evaluate the degree of sequence conservation between *B*. *humilis* and *A*. *thaliana*. The results of this comparison revealed that 31,641 of the 46,485 *B*. *humilis* unigenes yielded best hits with *A*. *thaliana* protein sequences; approximately 85% share at least 70% similarity with *A*. *thaliana* proteins, while 35% exhibit 70% coverage compared to an *A*. *thaliana* protein (Fig 2A and 2B). A smooth scatter plot shows that 9,785 *B*. *humilis* unigenes match with an *A*. *thaliana* protein with more than 70% similarity and coverage, while 21,856 match with less than 70% similarity and coverage (Fig 2C). These results suggest that our *B*. *humilis* transcriptome dataset has good coverage of homologs in its relative *A*. *thaliana* and can thus provide sufficient information for the subsequent analysis of conserved genes in this species.

**Fig 3:**
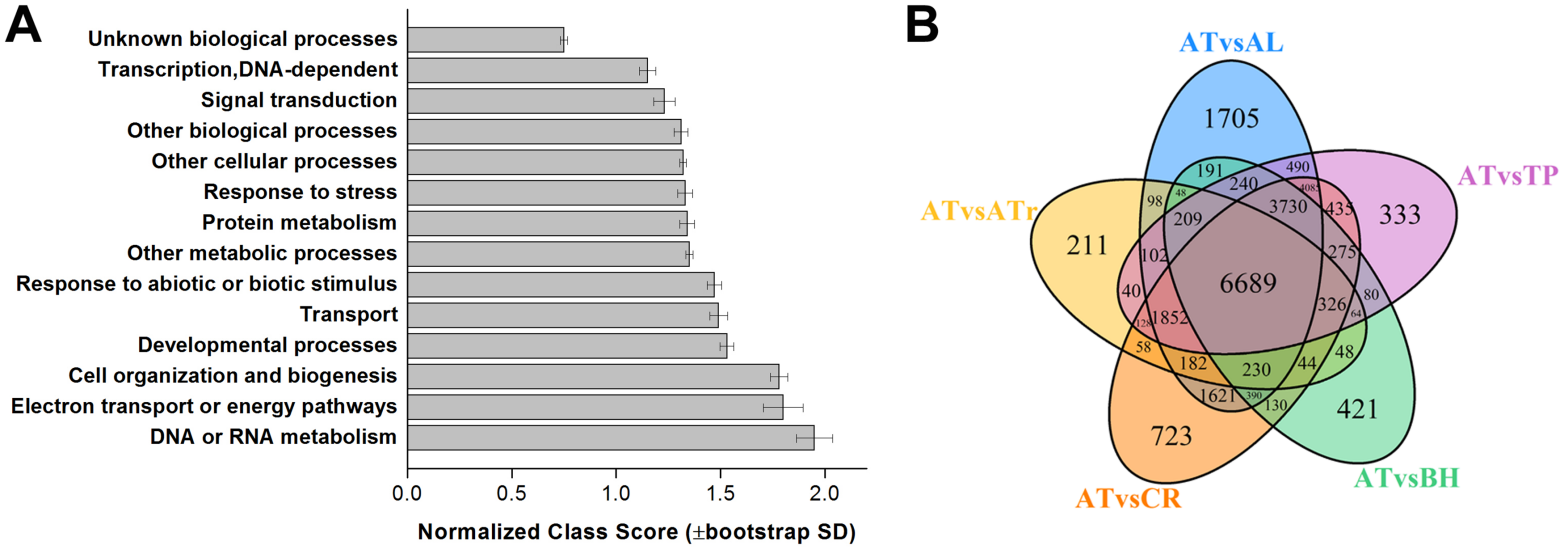
Results of GO enrichment analyses of 6,689 conserved genes in *B*. *humilis*. GO enriched terms (*p* < 0.01) were analyzed using the Classification SuperViewer Tool and normalized (A) results are presented. (B) Venn diagram representation to show the conserved genes between *B*. *humilis* and the other five other sequenced species. Abbreviations: AT, *A*. *thaliana*; AL, *A*. *lyrata*; CR, *C*. *rubella*; TP, *T*. *parvula*; ATr, *A*. *trichopoda*.

**Fig 4:**
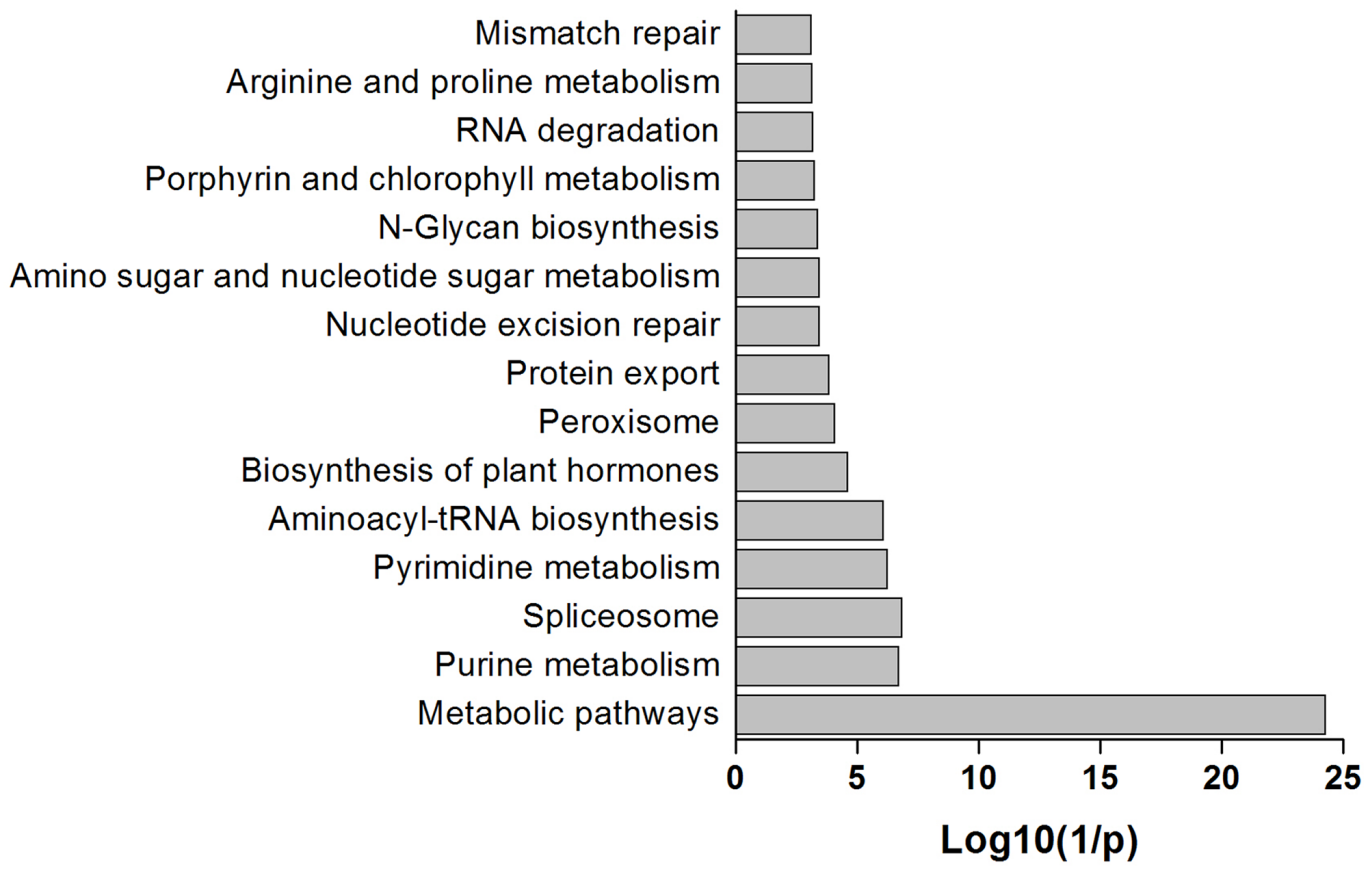
Results of KEGG pathway enrichment analyses of 6,689 orthologous genes in *B*. *humilis*. KEGG pathway enrichment was analyzed using the PlantGSEA program and the cutoff value of FDR was 0.05.

To further investigate the relationship between *B*. *humilis* and other species within the Brassicaceae as well as to explore core orthologous genes, we constructed a RBH Blast program using an e-value threshold of 1e-10 to compare *A*. *thaliana* genes with *B*. *humilis*, *A*. *lyrata*, *C*. *rubella*, and *T*. *parvula* genes. This approach led to the generation of four sets of RBHs with gene pair numbers ranging from 13,114 (*B*. *humilis*) to 21,994 (*A*. *lyrata*) between *A*. *thaliana* and the other four species of Brassicaceae (S3 Table). In particular, *A*. *trichopoda* is the sole surviving sister species of all other extant flowering plants, the genome of this species provides a key reference for comparisons with other angiosperms [14,15]. We also included *A*. *trichopoda* in RBH analyses in order to precisely characterize the orthologous gene composition in *B*. *humilis*. Our results show that 10,328 *A*. *trichopoda* genes received best hits (e-value less than 1e-5) versus *A*. *thaliana* proteins. We then isolated the genes shared among all RBH results using the ‘VennDiagram’ function in R [17], with those common to all six species identified as essential orthologous *B*. *humilis* genes (Fig 3). This approach reveals that 6,689 genes are shared between *B*. *humilis* and the five other species (i.e. *A*. *thaliana*, *A*. *lyrata*, *C*. *rubella*, *T*. *parvula*, and *A*. *trichopoda*), while GO enrichment analyses using the Classification SuperViewer indicates that a range of highly conserved BP terms, including ‘DNA or RNA metabolism’, ‘electron transport or energy pathways’, ‘cell organization and biogenesis’, ‘developmental process’, and ‘protein metabolism’, are overrepresented (Fig 3). These results demonstrate the conservation of numerous fundamental BPs during the evolution of *B*. *humilis* and other plant species. KEGG enrichment analysis using the PlantGSEA identified fifteen representative pathways (FDR < 0.05) and pathways, including ‘biosynthesis of plant hormones’ and ‘peroxisome’, are specifically enriched (FDR < 0.01; Fig 4). We then applied stricter thresholds for e-values, hit lengths on an *A*. *thaliana* peptide, and identity values to further reduce the list to screen for core orthologous genes. Results reveal (S4 Table) the identification of 4,539 genes even given the application of e-values less than 1e-40, hit lengths greater than, or equal to, 100 amino acids, and identity values greater than, or equal to, 50%. This enabled us to identify a core set of 381 genes with e-values less than 1e-40, hit lengths greater than, or equal to, 100 amino acids, and identities greater than, or equal to, 80% as the key genes shared between *B*. *humilis* and the five other species. Our annotation pathway also shows that most GO terms, with the exception of ‘DNA or RNA metabolism’, ‘signal transduction’, and ‘transcription’, are consistently enriched in all categories given different selection thresholds (S3 Fig). Enriched stress-related GO terms suggest that some of the same genetic modules are used by *B*. *humilis* in its adaptations to arid environments.

### Core salt and osmotic stress responsive genes in *B*.*humilis*

**Fig 5:**
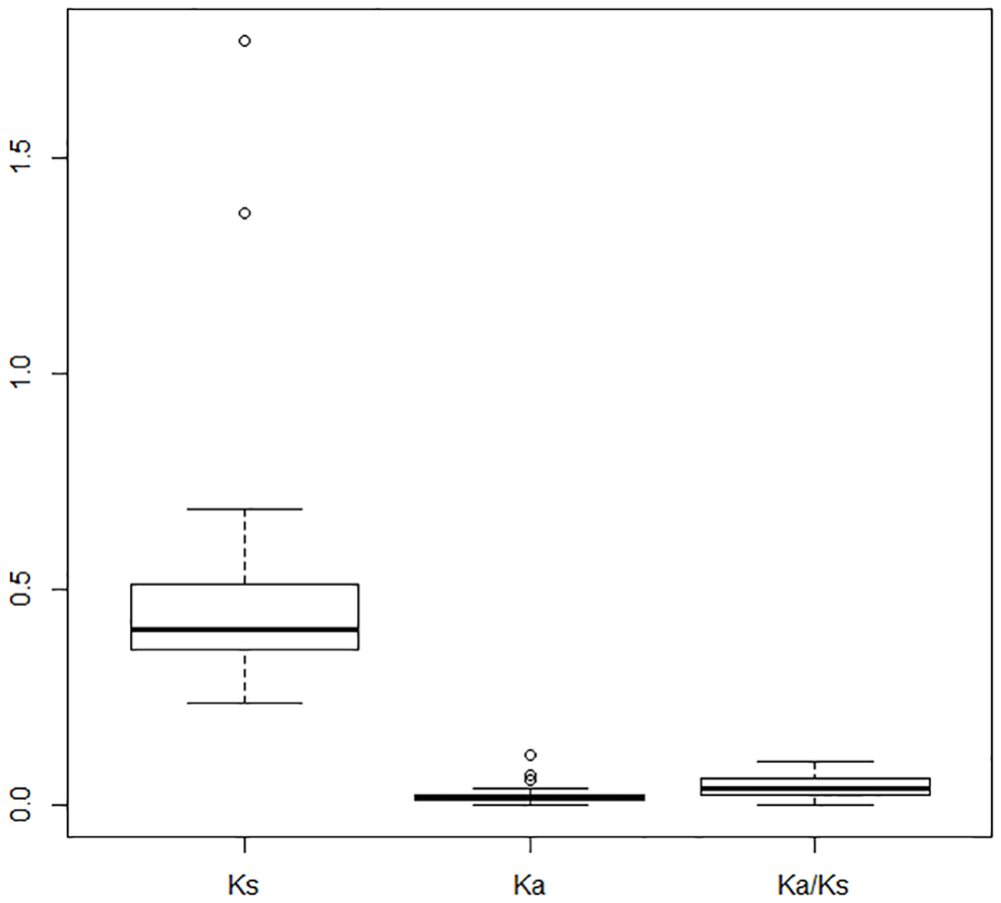
Ka/Ks results of the 39 salt and osmotic stress genes in *B*. *humilis* versus *A*. *thaliana* orthologs.

**Fig 6:**
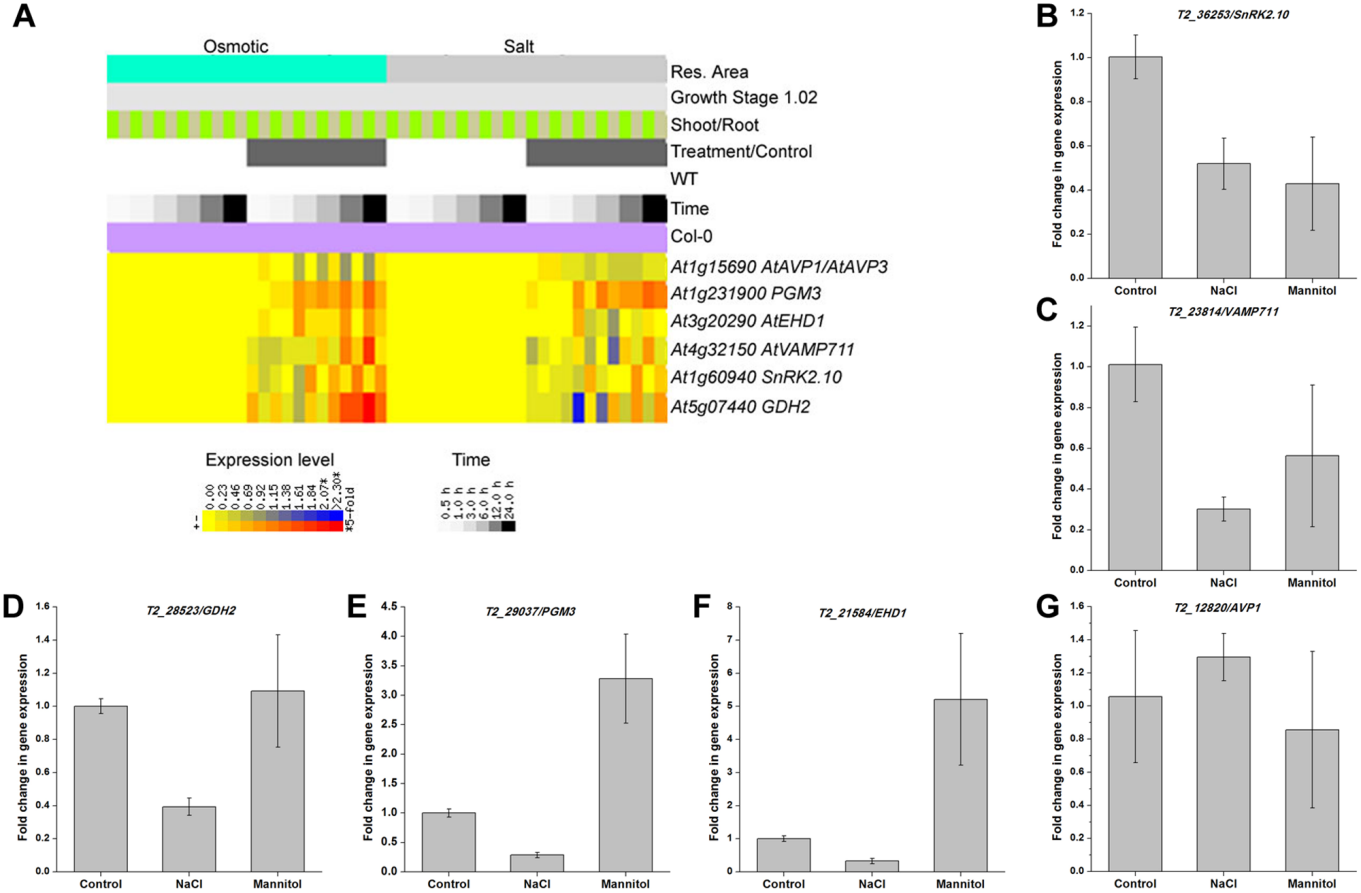
Expression patterns of the six core stress responsive genes in *B*. *humilis*. (A) Heatmap to show six *Arabidopsis* gene expression profiles under osmotic and salt stress treatments. This graphic was created using the BAR Expression Browser [31]. The expression profiles of these six genes in response to other abiotic stresses are shown in Supplementary Fig 4. (B) The transcript levels of six core stress genes in *B*. *humilis* seedlings were determined after treatments with 150 mM NaCl and 300 mM mannitol. Error bars represent ± SE.

The Ka/Ks ratio is one useful index that can be used to illustrate evolutionary patterns in orthologous genes between different plant species [27–30]. Results show that 39 of the 381 core orthologous genes identified in this study were assigned functions related to salt and osmotic stress responses as well as water deprivation (S5 Table). Ka/Ks ratio estimations of these *B*. *humilis* genes versus their counterparts in *A*. *thaliana* reveal that all these ratios are less than 1 (Fig 5), which suggests that these genes were subject to purifying selection during *B*. *humilis* speciation. We then generated a heat map of the 39 gene expression signal intensities using the BAR Expression Browser [31] based on *A*. *thaliana* (growth stage 1.02; [32]) transcriptome data from cold, drought, heat, osmotic, oxidative, and salt stress treatments to obtain further insights into expression profiles following abiotic stress (Fig 6 and S4 Fig). These results show that expression of the *heat shock cognate 70–5* (*At5g09590*) gene was significantly induced by osmotic, salt, and heat stress treatments, while the *light-harvesting chlorophyll B-binding 2* (*At3g27690*) gene was expressed to a high level under cold stress conditions and to a lesser extent under osmotic stress. AVP1 is a vacuolar proton-pumping pyrophosphatase; although overexpression of the *AVP1* (*At1g15690*) gene in plants confers high tolerance against drought and salinity stress [33], its transcription levels changed only slightly, while the *SNF1-related protein kinase 2*.*10* (*At1g60940*, *SnRK2*.*10*), *phosphoglucomutase* 3 (*At1g23190*, PGM3), *EPS15 homology domain 1* (*At3g20290*, *EHD1*), *vesicle-associated membrane protein 711* (*At4g32150*, *VAMP711*), and *glutamate dehydrogenase 2* (*At5g07440*, *GDH2*) genes all showed high levels of expression after 24 hours of osmotic stress.

The species *B*. *humilis* is thought to be abiotic stress tolerant because of the Central Asia habitats it occupies, characterized by low precipitation, an arid climate, and a saline environment [34]. Our previous research has shown that *B*. *humilis* plants only wither at their leaf tips following 12 hours of 40% polyethylene glycol 6000 treatment [35]. Thus, in order to investigate the functional conservation of these 39 core salt and osmotic genes in *B*. *humilis*, we further characterized the expression patterns of the six genes in seedlings following salt and mannitol treatments (Fig 6). These results show that the abundance of *SnRK2*.*10* and *VAMP711* gene transcripts was reduced as the result of salt (150 mM) and mannitol (300 mM) treatments for three hours, while the expression levels of *GDH2*, *PGM3*, and *EHD1* were suppressed by NaCl treatment, and *PGM3* and *EHD1* gene transcripts were induced by mannitol treatment to approximately three and five times control base levels. Results also show no significant difference in *AVP1* expression levels following salt and osmotic stress treatments compared to the control. The expression profiles of these six genes in *B*. *humilis* seedlings are highly consistent with those in *A*. *thaliana* (Fig 6 and S4 Fig).

## Conclusions

The Brassicaceae species *B*. *humilis* is a widely adapted to a range of different environmental conditions. We assembled and annotated a highly accurate transcriptome for this plant in this study and conducted a series of comparative transcriptomic analyses that reveal a significant amount of gene conservation between *B*. *humilis* and *A*. *thaliana*. We also identify a series of orthologous genes within five species of Brassicaceae and *A*. *trichopoda*. As the results of this study show that the core salt and osmotic stress response genes in *B*. *humilis* have been subject to purifying selection during evolution and exhibit similar patterns of expression to their counterparts in *Arabidopsis*, it is likely that some of the same genetic components have been employed in *B*. *humilis* to confer environmental adaptations. Additional research will be required to gain further insights on the mechanisms of adaptation of *B*. *humilis*; these core stress genes should be subject to further analysis in natural populations to determine whether, or not, their expression profiles are correlated with local climate change.

## Supporting information

S1 Fig Verification of the quality of unigene assembly using RT-PCR. Twenty-four unigenes were randomly selected for verification using RT-PCR and 5 μl of PCR products were loaded. Abbreviation: M, marker 3.
(TIF)

S2 Fig Hit characteristics of *B*. *humilis* unigenes versus sequences deposited in the Nr database. (A) Nr annotation results illustrated using e-values. (B) Nr annotation results based on sequence identities.
(TIF)

S3 Fig Results of GO enrichment analyses of the 381 orthologous *B*. *humilis* genes. Normalized results (A) of GO enriched terms (*p* < 0.01) are presented in this figure. (B) The 381 core orthologous genes shared between *B*. *humilis* and the other five sequenced species.
(TIF)

S4 Fig The expression profiles of 39 core stress genes in *Arabidopsis* seedlings following different abiotic stress treatments. These microarray data are from the AtGenExpress Consortium, and the heatmap was generated using BAR Expression Browser [31]. This graphic also includes six genes from Fig 6.
(TIF)

S1 Table Overview of the *de novo* sequence assembly process for *B*. *humilis*. (DOCX)

S2 Table Summary of GO annotations for the *B*. *humilis* transcriptome. (XLSX)

S3 Table RBH results. (XLSX)

S4 Table Orthologous genes with different selection thresholds. (DOCX)

S5 Table The 39 core salt and osmotic stress genes in *B*. *humilis* and GO annotation. (XLSX)
