# Supplementary figures and images for: A comparative transcriptomic analysis reveals the core genetic components of salt and osmotic stress responses in Braya humilis
Source: PLoS One. 2017 Aug 31;12(8):e0183778. doi: 10.1371/journal.pone.0183778 (PMC5578489; doi:10.1371/journal.pone.0183778)

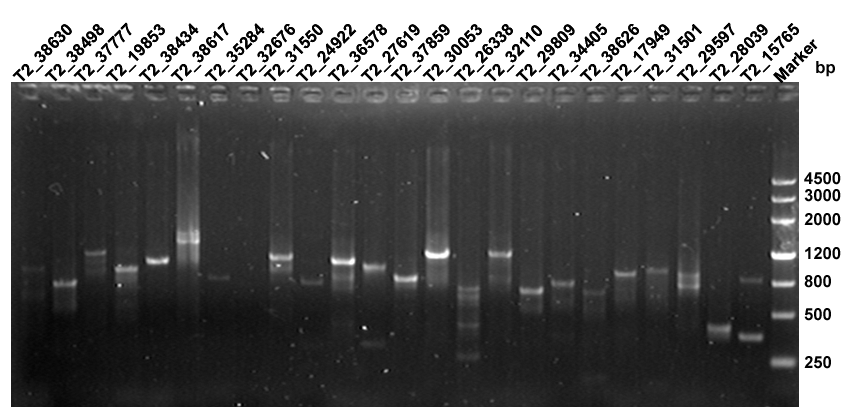

Supplement: S1 Fig — Twenty-four unigenes were randomly selected for verification using RT-PCR and 5 μl of PCR products were loaded. Abbreviation: M, marker 3. (TIF) [file pone.0183778.s001.tif]

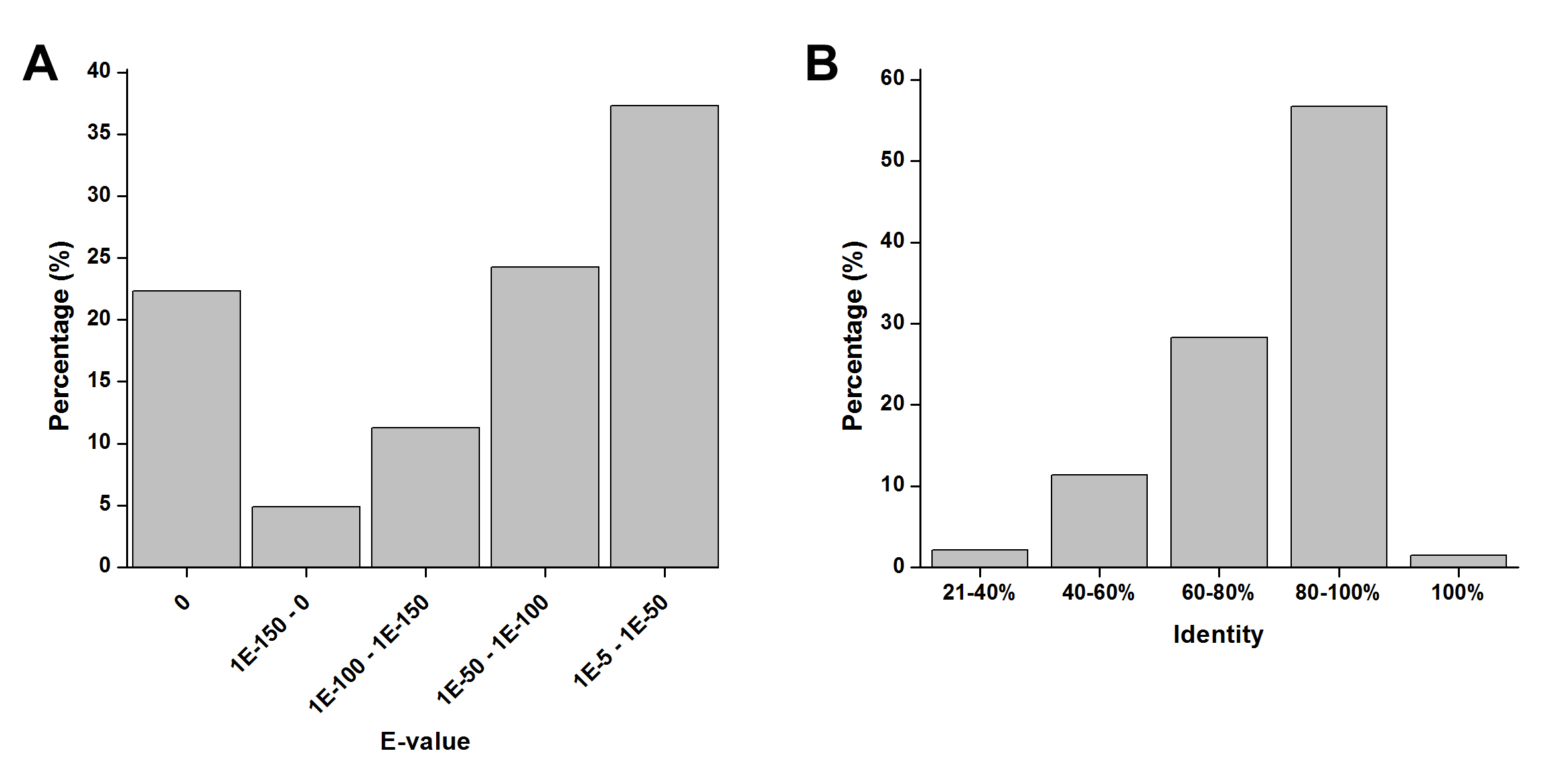

Supplement: S2 Fig — (A) Nr annotation results illustrated using e-values. (B) Nr annotation results based on sequence identities. (TIF) [file pone.0183778.s002.tif]

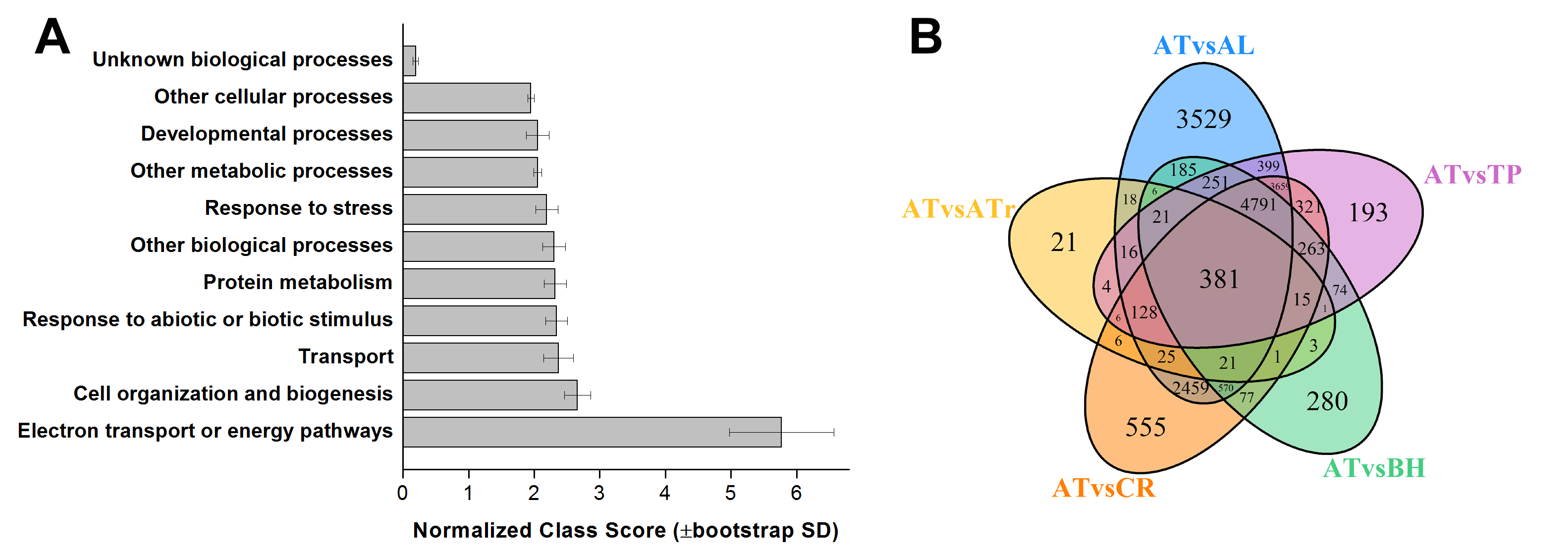

Supplement: S3 Fig — Normalized results (A) of GO enriched terms (p < 0.01) are presented in this figure. (B) The 381 core orthologous genes shared between B. humilis and the other five sequenced species. (TIF) [file pone.0183778.s003.tif]

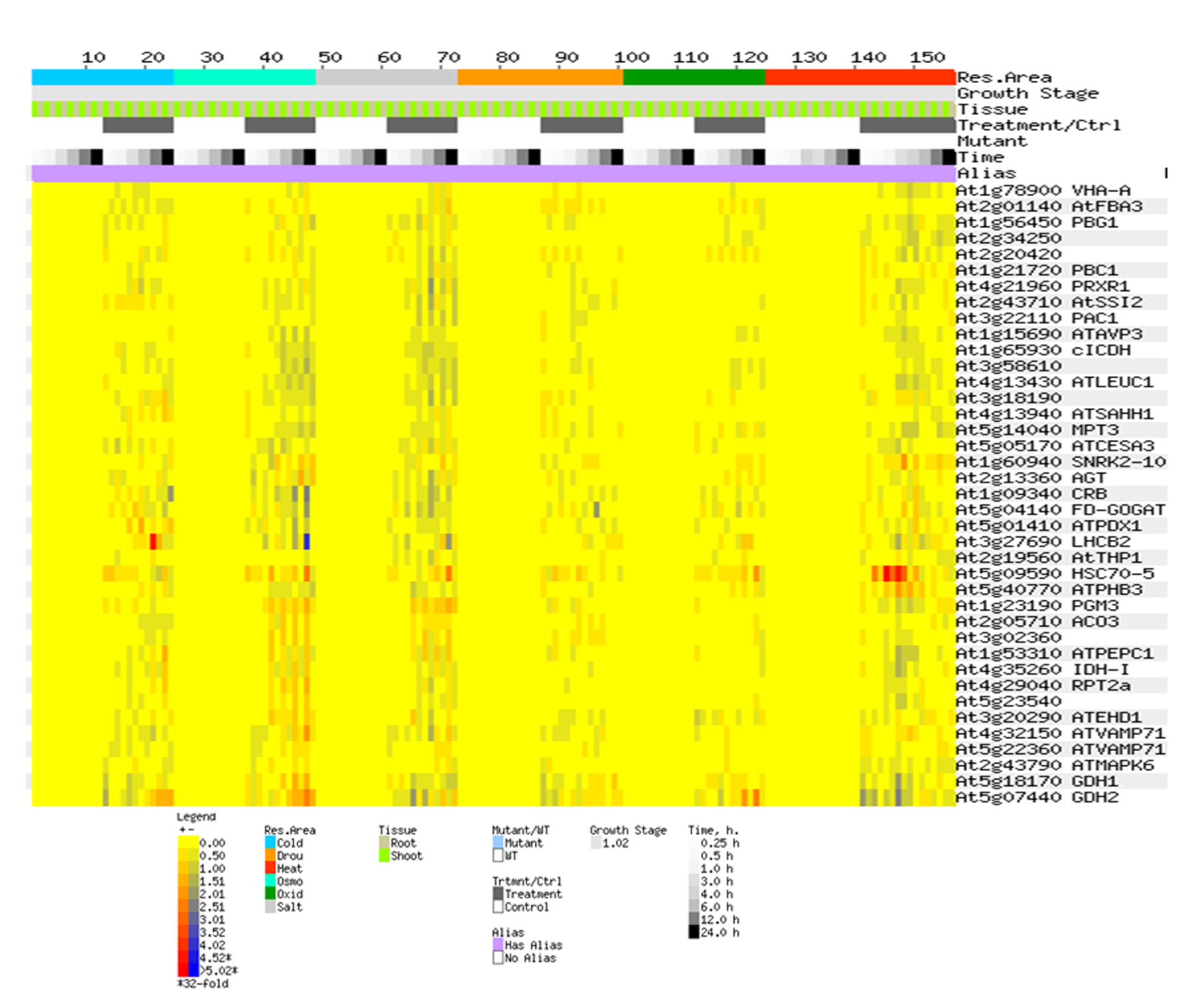

Supplement: S4 Fig — These microarray data are from the AtGenExpress Consortium, and the heatmap was generated using BAR Expression Browser [31]. This graphic also includes six genes from Fig 6. (TIF) [file pone.0183778.s004.tif]
