# Supplementary material for: A comparative transcriptomic analysis reveals the core genetic components of salt and osmotic stress responses in Braya humilis
Source: PLoS One. 2017 Aug 31;12(8):e0183778. doi: 10.1371/journal.pone.0183778 (PMC5578489; doi:10.1371/journal.pone.0183778)
Supplement: S1 Table — (DOCX) [file pone.0183778.s005.docx]

**Supplementary Table 1** Overview of the *de novo* sequence assembly process for *B. humilis*.

| Length range (bp) | Contigs | Transcripts | Unigenes |
| --- | --- | --- | --- |
| 0-300 | 2,339,649 (98.38%) | 18,441 (19.50%) | 13,602 (29.26%) |
| 300-500 | 15,069 (0.63%) | 19,426 (20.54%) | 11,922 (25.65%) |
| 500-1000 | 11,539 (0.49%) | 23,943 (25.32%) | 9,724 (20.92%) |
| 1000-2000 | 8,398 (0.35%) | 23,044 (24.37%) | 7,964 (17.13%) |
| >2000 | 3,618 (0.15%) | 9,708 (10.27%) | 3,273 (7.04%) |
| Total number | 2,378,273 | 94,562 | 46,485 |
| Total length | 157,294,809 | 90,373,588 | 35,529,081 |
| N50 length | 58 | 1,437 | 1,235 |
| Mean length | 66.14 | 955.71 | 764.31 |
