# Supplementary material for: A comparative transcriptomic analysis reveals the core genetic components of salt and osmotic stress responses in Braya humilis
Source: PLoS One. 2017 Aug 31;12(8):e0183778. doi: 10.1371/journal.pone.0183778 (PMC5578489; doi:10.1371/journal.pone.0183778)
Supplement: S4 Table — (DOCX) [file pone.0183778.s008.docx]

**Table S4** Orthologous genes with different selection thresholds.

| Cluster | At vs ATr RBHs | | | Other four pairs of RBHs | | | Number of shared genes |
| --- | --- | --- | --- | --- | --- | --- | --- |
|  | E value | Hit length (aa) | Identity (%) | E value | Hit length (aa) | Identity (%) |  |
| 1# | 1e-5 |  |  | 1e-10 |  |  | 6689 |
| 2# | 1e-10 | 50 |  | 1e-20 | 50 |  | 6593 |
| 3# | 1e-10 | 100 |  | 1e-20 | 100 |  | 6177 |
| 4# | 1e-20 | 100 |  | 1e-20 | 100 |  | 6072 |
| 5# | 1e-20 | 100 |  | 1e-20 | 100 | 50 | 6052 |
| 6# | 1e-20 | 100 |  | 1e-40 | 100 | 50 | 6019 |
| 7# | 1e-40 | 100 |  | 1e-40 | 100 | 50 | 5658 |
| 8# | 1e-40 | 100 | 50 | 1e-40 | 100 | 50 | 4539 |
| 9# | 1e-40 | 100 | 50 | 1e-40 | 100 | 70 | 4263 |
| 10# | 1e-40 | 100 | 60 | 1e-40 | 100 | 80 | 2449 |
| 11# | 1e-40 | 100 | 70 | 1e-40 | 100 | 80 | 1289 |
| 12# | 1e-40 | 100 | 80 | 1e-40 | 100 | 80 | 381 |
